# Inflammation–fibrosis crosstalk in endometriosis: immune–stromal mechanisms linking lesion persistence to pain and infertility

**DOI:** 10.3389/fmed.2026.1984591

**Published:** 2026-09-30

**Authors:** Xiaofeng Liu, Zheng Qu, Jinhong Gu, Fang Lian, Shuguang Fu

**Affiliations:** 1 First College of Clinical Medicine, Shandong University of Traditional Chinese Medicine, Jinan, Shandong, China; 2 Qingdao Traditional Chinese Medicine Hospital, Qingdao Hiser Hospital Affiliated of Qingdao University, Qingdao, Shandong, China; 3 Department of Reproductive and Genetics Medicine, Affiliated Hospital of Shandong University of Traditional Chinese Medicine, Jinan, Shandong, China

**Keywords:** endometriosis, fibrosis, immune–stromal crosstalk, infertility, inflammation, pelvic pain

## Abstract

Endometriosis is an estrogen-dependent chronic inflammatory disorder in which ectopic endometrial-like tissue undergoes repeated injury, incomplete resolution, and progressive remodeling. Fibrosis is therefore not merely a terminal scar but an active component of lesion biology. Cyclic bleeding activates platelets and innate immune cells, generates iron-dependent oxidative stress and hypoxia, and sustains transforming growth factor-β, sphingosine-1-phosphate, Wnt/β-catenin, PI3K/AKT–ERK, and Notch-related signals. These cues promote fibroblast activation, myofibroblast differentiation, extracellular-matrix deposition, and epithelial-, endothelial-, mesothelial-, or macrophage-to-mesenchymal plasticity. The resulting stiff matrix can further reinforce inflammatory, vascular, and neural programs, creating a self-maintaining inflammation–fibrosis circuit. Clinically, this circuit may stabilize ectopic lesions, promote adhesions, increase tissue tension and neurogenic inflammation, damage the ovarian cortex, and perturb the peritoneal and eutopic endometrial environments. These effects provide mechanistic links to persistent pain and infertility, although fibrosis is neither the sole determinant of symptoms nor a uniform feature across lesion phenotypes. This Mini Review integrates evidence from experimental models, human pathology, and single-cell studies; identifies uncertainties in cellular lineage, lesion chronology, and outcome attribution; and discusses therapeutic implications. Conventional hormonal and surgical treatment remains central, whereas antifibrotic, immunomodulatory, and complementary approaches require mechanism-based clinical validation. Progress will depend on standardized fibrosis phenotyping, longitudinal and spatial sampling, patient-derived multicellular models, and trials that align biological targets with pain, fertility, and recurrence outcomes.

## 1 Introduction

Endometriosis is defined by endometrial-like glands and stroma outside the uterine cavity and is commonly associated with dysmenorrhea, chronic pelvic pain, dyspareunia, subfertility, and impaired quality of life (1–4). Contemporary guidelines emphasize individualized combinations of analgesia, hormonal suppression, surgery, and fertility-directed care, but no current strategy reliably eradicates the disease or prevents all symptom recurrence (5). These limitations have intensified interest in the tissue ecology that allows lesions to survive after retrograde menstruation or other initiating events.

Inflammation has long been central to endometriosis pathophysiology, but inflammation alone does not explain why lesions become firm, adherent, deeply infiltrative, or anatomically destructive. Fibrosis—the excessive accumulation and remodeling of extracellular matrix (ECM)—is increasingly recognized as an intrinsic disease process rather than an incidental consequence of chronicity (6–9). Collagen deposition, smooth-muscle metaplasia, activated fibroblasts, myofibroblasts, and increased tissue stiffness are found to varying degrees in ovarian, peritoneal, and deep lesions. Importantly, fibrosis can also modify cell behavior: a stiff matrix changes mechanosignaling, diffusion, vascular organization, and nerve–matrix interactions, potentially feeding back on inflammation and lesion persistence.

Fibrosis is also heterogeneous. Superficial peritoneal implants may contain small, intermittently active fibrotic foci; ovarian endometriomas combine a fibrotic pseudocapsule with damage to adjacent cortex; and deep lesions often contain abundant smooth-muscle-like tissue, dense collagen, and organ-specific neural and vascular elements. These phenotypes should not be placed on a single universal timeline. Some lesions may progress from red, vascular, inflammatory implants toward pigmented and fibrotic forms, whereas others may arise or persist through distinct tissue programs. A useful model must therefore explain a shared injury–repair logic without assuming that every lesion follows the same cellular route or produces the same clinical outcome. In practical terms, superficial peritoneal lesions often show more limited or focal fibrosis, ovarian endometriomas combine pseudocapsular fibrosis with adjacent cortical injury, and deep endometriosis more often exhibits dense collagen and smooth-muscle-like remodeling; substantial heterogeneity nevertheless remains within each phenotype.

This Mini Review advances a focused model in which repeated tissue injury is converted into a self-reinforcing inflammation–fibrosis circuit through immune–stromal crosstalk. We examine how platelets, macrophages, lesion epithelial and stromal cells, fibroblasts, vascular cells, and sensory nerves participate; how major signaling modules converge on matrix-producing cells; and how the resulting remodeling may contribute to pain and infertility. We also distinguish mechanistic plausibility from established clinical causality, because lesion fibrosis, symptom severity, and reproductive outcomes do not map onto one another in a simple linear manner.

A focused literature search was conducted in PubMed, Web of Science, and Google Scholar from database inception through September 2026 using combinations of “endometriosis,” “fibrosis,” “inflammation,” “immune,” “stromal,” “TGF-β,” “pain,” and “infertility.” Priority was given to human studies, mechanistic investigations, systematic reviews, and recent single-cell or spatial studies, with seminal experimental studies included when needed to define established mechanisms.

## 2 Cyclic injury converts unresolved inflammation into fibrosis

Fibrosis is best understood as dysregulated wound repair. In normal repair, inflammatory recruitment, fibroblast activation, provisional matrix deposition, and matrix resolution occur in a temporally coordinated sequence. Persistent injury or defective resolution instead maintains myofibroblasts and shifts ECM turnover toward accumulation, crosslinking, and architectural distortion (10, 11). Endometriotic lesions are unusually suited to this transition because hormonally responsive tissue can bleed repeatedly in an ectopic compartment that lacks efficient drainage and physiological endometrial regeneration.

Cyclic hemorrhage supplies heme, free iron, reactive oxygen species, cellular debris, and damage-associated signals. These stimuli activate mesothelial and endothelial surfaces, recruit innate immune cells, and create hypoxic and oxidative conditions that support angiogenesis and inflammatory survival programs. Transforming growth factor-beta (TGF-β) is a central bridge between these processes: it influences immune-cell behavior, stromal proliferation, epithelial and mesenchymal plasticity, matrix synthesis, and protease regulation in peritoneal endometriosis (12). The pathway is not isolated; its fibrogenic output depends on cellular source, receptor context, lesion site, and the mechanical state of the surrounding matrix.

Platelets provide a particularly direct link between repeated bleeding and fibrosis. Activated platelets accumulate around damaged microvessels and release TGF-β, platelet-derived growth factors, chemokines, and lipid mediators. Experimental studies indicate that platelet signaling can promote epithelial-mesenchymal transition-like changes, fibroblast-to-myofibroblast transdifferentiation, smooth-muscle metaplasia, and endothelial-mesenchymal transition, while antiplatelet intervention can restrain lesion progression in induced disease models (13–15).

Human and animal data further implicate TGF-β/SMAD signaling in ovarian and peritoneal fibrosis. TGF-β1 derived from endometrioma-related tissue can activate SMAD2/3 and promote fibrotic remodeling in adjacent ovarian cortex, whereas host TGF-β1 deficiency suppresses lesion development in mice (16, 17). Downstream and parallel pathways expand this response. Canonical Wnt/β-catenin signaling cooperates with TGF-β to sustain fibroblast activation, and endometriosis-derived mesenchymal stromal cells can reinforce this program through paracrine TGF-β1 and Wnt1 (18, 19). Oxidative stress-associated ADAM17/Notch dysregulation, coordinated AKT and ERK activation, and sphingosine-1-phosphate (S1P) signaling have also been linked to fibrogenic behavior (20–23). Together, these data favor a network model rather than a single-master-pathway model: repeated bleeding initiates overlapping inflammatory, growth-factor, metabolic, and mechanosensitive signals that converge on matrix-producing cells (Figure 1).

**Figure 1:**
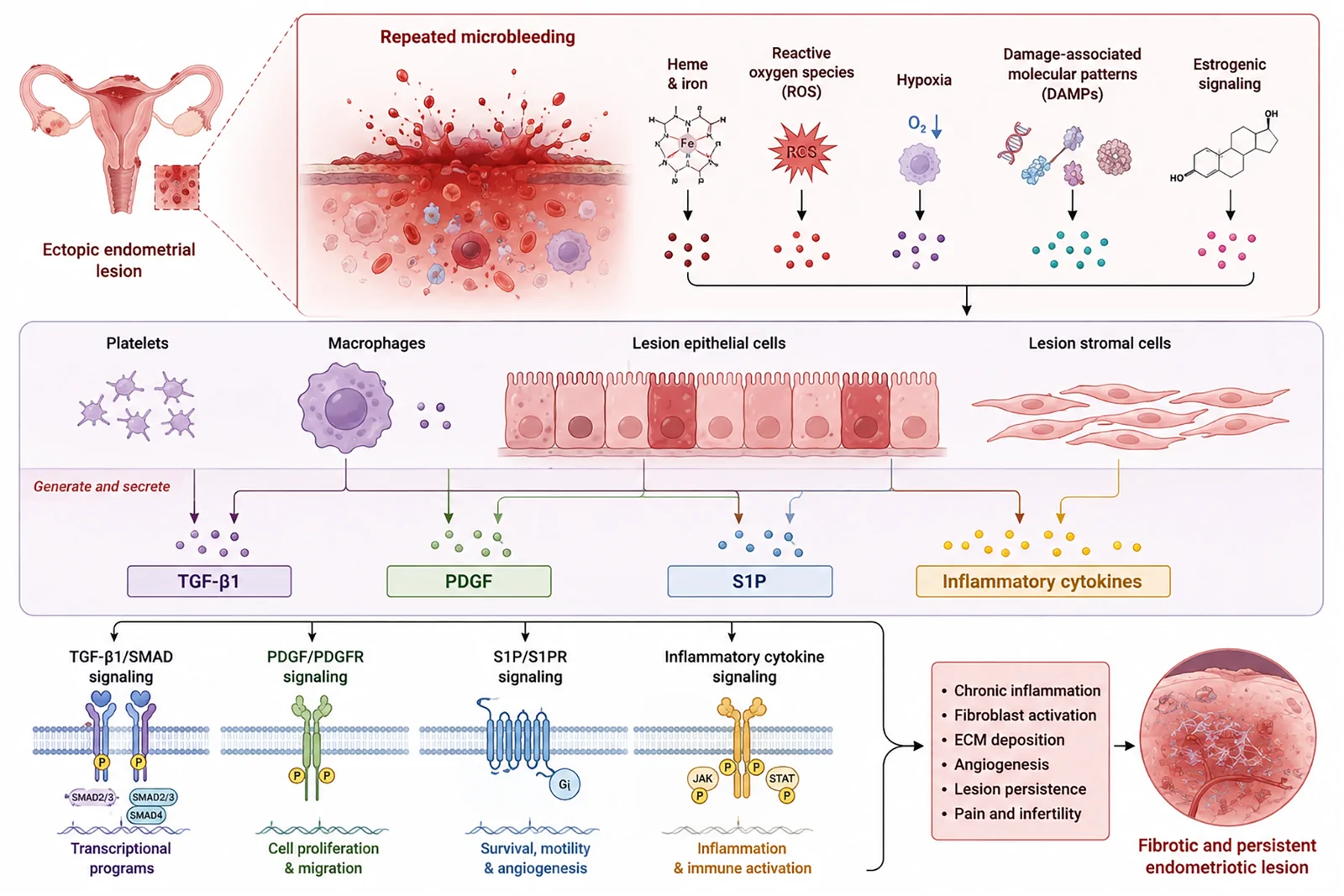
Cyclic injury establishes a self-reinforcing inflammation–fibrosis circuit in endometriosis. Repeated microbleeding exposes ectopic tissue to heme and iron, reactive oxygen species, hypoxia, damage-associated molecular patterns, and estrogenic signaling. Platelets, macrophages, and lesion epithelial and stromal cells generate TGF-β1/SMAD, PDGF, S1P, and inflammatory cytokine signals. ROS, reactive oxygen species; DAMPs, damage-associated molecular patterns; TGF-β1, transforming growth factor-β1; PDGF, platelet-derived growth factor; S1P, sphingosine-1-phosphate; ECM, extracellular matrix; EMT, epithelial-mesenchymal transition; EndoMT, endothelial-mesenchymal transition; MMT, macrophage-myofibroblast transition.

The fibrotic product is itself biologically active. Activated fibroblasts and myofibroblasts deposit collagens, fibronectin, proteoglycans, and matricellular proteins while altering matrix metalloproteinase and tissue-inhibitor activity. Enzymatic crosslinking and contractility increase tissue stiffness and reduce reversibility even when the original inflammatory stimulus fluctuates. Stiffness can enhance integrin–focal adhesion signaling, cytoskeletal tension, and nuclear mechanotransduction, thereby maintaining survival and matrix synthesis. At the same time, dense ECM changes oxygen diffusion, interstitial flow, and the spatial access of immune cells. These feedbacks offer a plausible explanation for why established lesions may remain anatomically fibrotic despite hormonal suppression, while still exhibiting episodic inflammatory activity. They also caution against using collagen abundance as the only measure of fibrosis; fiber organization, crosslinking, stiffness, contractility, and active matrix turnover may be equally relevant.

## 3 Immune–stromal crosstalk determines fibrotic trajectory

Macrophages occupy a strategic position in the endometriotic niche because they sense blood products and damaged tissue, regulate angiogenesis, clear debris, and instruct fibroblasts. In mouse endometriosis, an M2a-like macrophage subset has been associated with fibrogenesis (24). More recent human and experimental evidence suggests that TGF-β1/SMAD3 signaling may also induce macrophage-to-myofibroblast transition, providing a potential direct source of matrix-producing cells (25). Earlier studies identified alternatively activated macrophages in patients and showed that macrophages are required for lesion growth and vascularization in models (26). However, macrophage effects depend on ontogeny and context: tissue-resident and recruited populations can exert opposing lesion-promoting or lesion-limiting functions (27). The conventional M1/M2 binary is therefore useful as shorthand but insufficient for therapeutic stratification.

The broader immune environment reinforces this complexity. Altered natural killer-cell function, T-cell states, mast-cell activation, neutrophil recruitment, complement activity, cytokine production, and impaired resolution have all been described in endometriosis (28–30). These components can affect fibrosis indirectly by changing TGF-β availability, oxidative stress, vascular permeability, matrix metalloproteinase activity, and fibroblast survival. Conversely, dense ECM and altered stiffness can restrict immune-cell movement, change antigen-presenting-cell behavior, and create spatially protected niches. Inflammation and fibrosis should therefore be viewed as mutually conditioning states rather than sequential, independent phases.

Single-cell studies have begun to resolve the stromal compartment that receives these signals. Distinct fibroblast states, developmental trajectories, and immune-cell compositions have been identified across eutopic and ectopic tissues (31). A separate single-cell atlas described coordinated programs of immune tolerance and angiogenesis across endometriosis tissues, emphasizing that epithelial, stromal, endothelial, and immune compartments are transcriptionally coupled (32). These datasets strengthen the immune–stromal model but also expose major limitations. Most studies are cross-sectional, lesion sampling is spatially incomplete, prior hormonal treatment is heterogeneous, and transcriptomic identity does not prove lineage conversion or matrix-producing function. Human lineage-tracing evidence for epithelial-, endothelial-, mesothelial-, or macrophage-derived myofibroblasts remains limited. Functional validation in spatially preserved tissue and patient-derived multicellular models is therefore essential.

## 4 Fibrotic remodeling links lesions to pain and infertility

Fibrosis may promote lesion persistence by creating a mechanically stable scaffold that supports residual epithelial and stromal cells, neovessels, immune niches, and recurrent microhemorrhage. Collagen accumulation and contraction can tether pelvic structures, produce adhesions, and make deep lesions progressively less compliant. Yet fibrosis should not be equated with irreversible inactivity: even collagen-rich lesions can contain metabolically active inflammatory, vascular, and neural microdomains. Lesion phenotype and age are likely to determine whether inflammation, fibrosis, or both dominate at a given time. Deep lesions involving bowel, bladder, uterosacral ligaments, or pelvic nerves may generate organ-specific consequences through smooth-muscle hyperplasia, luminal narrowing, local denervation or hyperinnervation, and traction on surrounding structures. Postoperative recurrence also has more than one meaning: a newly visible lesion, regrowth of residual disease, and return of pain without imaging recurrence may reflect different balances of inflammation, fibrosis, and neural sensitization.

Pain arises from a network extending beyond lesion volume. Menstruation-related injury can repeatedly trigger inflammatory and nociceptive signaling (33). Substance P and calcitonin gene-related peptide (CGRP), released from sensory fibers, can accelerate lesion development and fibrogenesis in experimental systems, while sensory nerve-derived neuropeptides can interact with inflammatory and stromal cells (34, 35). Neurogenic inflammation, mast-cell and macrophage activation, local estrogen signaling, nerve sprouting, and peripheral sensitization may then converge with central pain amplification (36, 37). Fibrosis may contribute by increasing tissue tension, entrapping or mechanically loading nerve fibers, and stabilizing neurovascular niches. However, pain intensity often diverges from anatomic stage, and persistent pain after technically successful surgery demonstrates that fibrosis is one component of a broader nociplastic and psychosocial system.

The fertility consequences are similarly compartment-specific (Figure 2). In ovarian endometriomas, iron-rich cyst fluid, oxidative stress, proteolytic activity, inflammation, and fibrotic replacement of cortex may reduce the functional follicular environment. Cortex adjacent to endometriomas shows enhanced follicular recruitment and atresia, while molecular and histological analyses support direct endometrioma-mediated damage beyond simple mechanical stretching (38, 39). Peritoneal inflammation can alter gamete transport, sperm function, oocyte competence, fertilization, and early embryo development; adhesions can distort tubo-ovarian anatomy; and surgery itself can reduce ovarian reserve if healthy cortex is removed or vascular supply is compromised (40).

**Figure 2:**
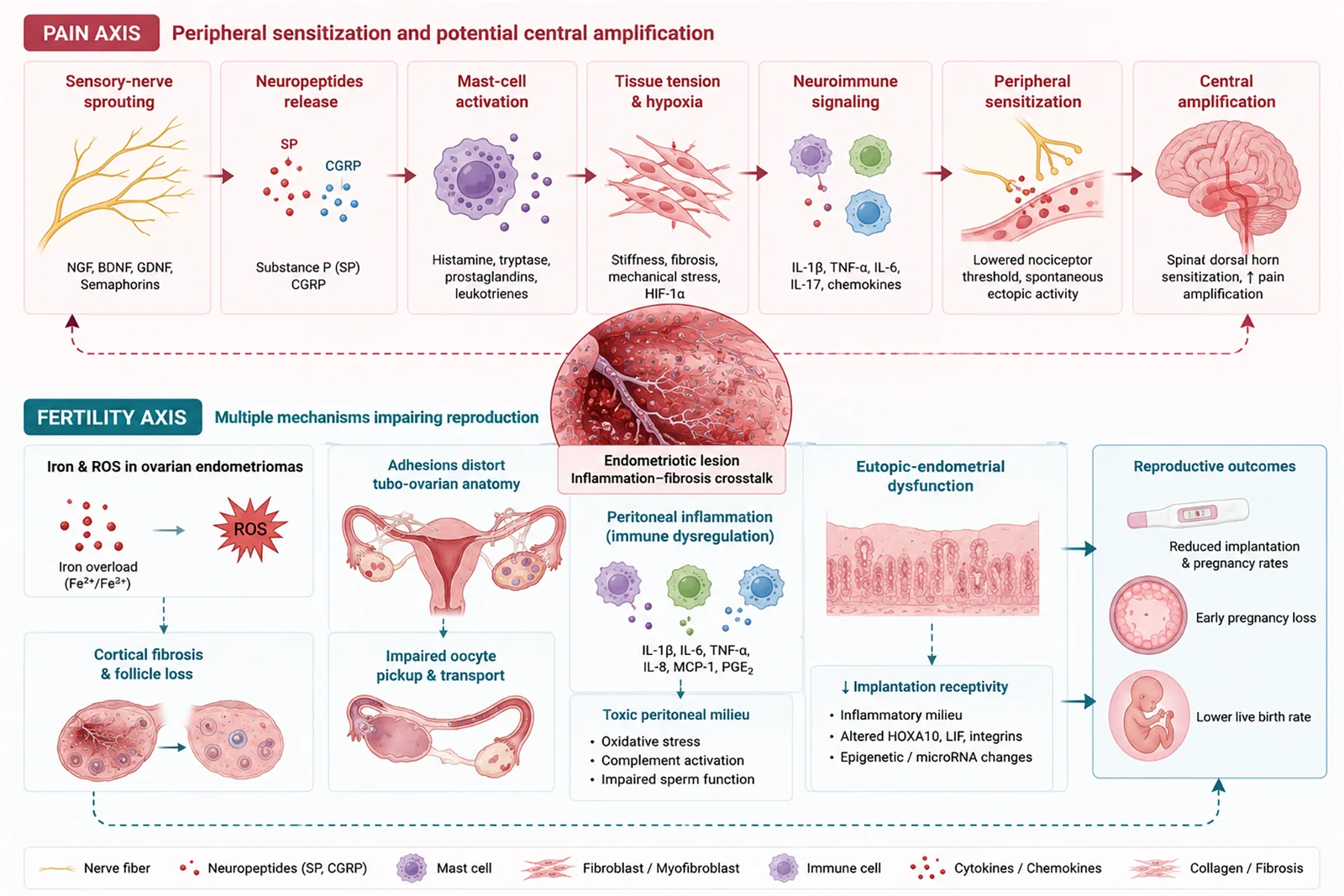
Spatial routes linking fibrotic endometriotic lesions to pain and infertility. The upper panel summarizes the pain axis, including neuroimmune signaling, tissue tension, peripheral sensitization, and potential central amplification. The lower panel summarizes the fertilit*y* axis, including ovarian cortical injury and fibrosis, adhesions, peritoneal inflammation, and eutopic-endometrial dysfunction.

At the uterine level, progesterone resistance, inflammatory signaling, altered decidualization, and impaired receptivity have been proposed in the eutopic endometrium (41). The magnitude and clinical relevance of these changes remain debated, particularly when euploid embryo transfer is considered. Reviews of *in vitro* fertilization outcomes likewise identify oxidative stress, steroidogenesis, inflammation, angiogenesis, and oocyte quality as plausible targets, but no antifibrotic intervention has yet been shown to consistently improve live birth (42). Thus, infertility cannot be reduced to lesion fibrosis alone; ovarian damage, pelvic anatomy, peritoneal biology, endometrial function, age, and prior treatment must be integrated. The relative contribution of fibrosis therefore varies among patients and should be interpreted alongside ovarian reserve, pelvic anatomy, age, inflammatory milieu, and prior treatment.

## 5 Therapeutic implications and a limited role for complementary approaches

Current care appropriately prioritizes evidence-based hormonal suppression, analgesia, fertility treatment, and expert surgery according to symptoms and reproductive goals (5). The inflammation–fibrosis model nevertheless suggests additional therapeutic principles. First, lesion stage may matter: early inflammatory lesions, mature collagen-rich lesions, and postoperative residual disease are unlikely to respond identically. Second, targeting one cytokine may fail because TGF-β/SMAD, Wnt/β-catenin, AKT/ERK, Notch, S1P, platelet, and macrophage programs are redundant and spatially compartmentalized. Third, systemic inhibition of core wound-healing pathways may impair host defense, vascular repair, implantation, or pregnancy. The most credible nonhormonal strategies will therefore need lesion-selective delivery, biomarker-defined populations, and explicit reproductive safety assessment.

Several intervention classes merit staged evaluation rather than immediate repurposing. Antiplatelet or S1P-directed approaches could interrupt blood-injury signaling, macrophage reprogramming could improve resolution, and blockade of TGF-β/Wnt or downstream fibroblast activation could reduce matrix production. Matrix-directed strategies might instead inhibit crosslinking or enhance controlled remodeling. Neuroimmune treatment may be required when pain persists after lesion-directed therapy. For each class, proof of target engagement should be demonstrated in lesion tissue or validated imaging biomarkers, not inferred solely from circulating cytokines. Trials should also separate prevention of new fibrosis from regression of established fibrosis and from symptomatic analgesia, because these are biologically different claims.

Traditional Chinese medicine can be discussed as a complementary, not disease-eradicating, option. A Cochrane review of Chinese herbal medicine found that available trials were small and methodologically weak, preventing firm conclusions regarding pain or fertility (43). A multicenter randomized, sham-controlled trial reported that acupuncture reduced endometriosis-associated pain and improved selected quality-of-life measures during the treatment period (44), and a subsequent meta-analysis suggested possible clinical benefit but highlighted heterogeneity, risk of bias, and limited durability data (45). Preclinical studies often attribute herbal or acupuncture effects to inflammatory, oxidative, neuroendocrine, or TGF-β-related pathways, but these mechanistic claims are not equivalent to demonstrated antifibrotic efficacy in human lesions. Such interventions may be considered adjunctively for symptom management when delivered safely and transparently, while high-quality trials should measure standardized pain outcomes, medication use, fertility endpoints, adverse events, and objective fibrosis markers.

## 6 Discussion and future directions

The inflammation–fibrosis explains several features of endometriosis: recurrent bleeding supplies injury signals; immune and platelet responses activate stromal cells; myofibroblasts remodel the ECM; and the remodeled matrix feeds back on inflammation, vascularization, and innervation. It also clarifies why hormonal suppression may reduce inflammatory activity without fully reversing established adhesions or deep fibrotic anatomy, and why surgery can remove fibrosis yet leave neural sensitization or microscopic disease.

Fibrosis is heterogeneous across superficial peritoneal, ovarian, and deep endometriosis; histological collagen abundance does not directly quantify stiffness, contractility, or biological activity; and cross-sectional specimens cannot reconstruct lesion chronology. Future studies should combine standardized histopathology with collagen architecture, ECM proteomics, biomechanical measurement, and imaging-based elastography. Sampling should record cycle phase, lesion subtype, pain phenotype, fertility status, prior surgery, and hormonal exposure. Longitudinal studies are needed to determine whether specific immune–stromal states predict lesion progression, recurrence, or treatment response.

Single-cell and spatial multi-omics should be linked to functional perturbation rather than used as descriptive endpoints. Organoids, stromal-immune co-cultures, microfluidic peritoneal models, and tunable three-dimensional matrices can test how cell identity changes across stiffness and inflammatory gradients. Candidate targets should then be assessed in models that preserve ovarian, neural, vascular, and implantation-related safety. Clinically, trials should distinguish symptom relief from biological modification and should not infer antifibrotic action from changes in serum cytokines alone. Recent work reinforces this direction: single-cell profiling has highlighted NK-cell dysfunction and immune dysregulation, integrative transcriptomic analysis has linked inflammatory signaling with intrinsic molecular programs, and methodological reviews have emphasized persistent translational gaps between experimental models and clinical endometriosis (46, 47).

In conclusion, fibrosis in endometriosis should be regarded as a dynamic, lesion-specific remodeling process driven by persistent immune–stromal interactions rather than as a passive end-stage scar. By altering extracellular-matrix architecture, vascular and neural niches, and reproductive tissue function, fibrotic remodeling may contribute to lesion persistence, pain, and infertility in a phenotype-dependent manner. Future studies should therefore focus on lesion-specific biomarkers and targeted interventions that distinguish active inflammation from established fibrosis while preserving reproductive function.
